# Alginate Encapsulation of *Begonia* Microshoots for Short-Term Storage and Distribution

**DOI:** 10.1155/2013/341568

**Published:** 2013-12-12

**Authors:** Hamidou F. Sakhanokho, Cecil T. Pounders, Eugene K. Blythe

**Affiliations:** ^1^USDA-ARS, Southern Horticultural Laboratory, 810 Highway 26 West, Poplarville, MS 39470, USA; ^2^Coastal Research and Extension Center, Mississippi State University, South Mississippi Branch Experiment Station, 810 Highway 26 West, Poplarville, MS 39470, USA

## Abstract

Synthetic seeds were formed from shoot tips of two *in vitro* grown *Begonia* cultivars using 3% sodium alginate in Murashige and Skoog medium (MS) salt solution as the gel matrix and 100 mM calcium chloride for complexation. Synthetic seed formation was achieved by releasing the sodium alginate/explant combination into 100 mM calcium chloride (CaCl_2_ *·*H_2_O) solution for 30 or 45 min. Both control and encapsulated shoots were transferred into sterile Petri dishes and stored at 4°C or 22°C for 0, 2, 4, 6, or 8 weeks. Conversion of synthetic seeds into plantlets for both storage environments was assessed in MS medium or peat-based substrate. No significant difference was found between the 30 and 45 min CaCl_2_ *·*H_2_O treatments or the two cultivars. Encapsulation of explants improved survival rate over time irrespective of the medium type or storage environment. Survival rates of 88, 53, 28, and 11% for encapsulated microshoots versus 73, 13, 0, and 0% for control explants were achieved in microshoots stored for 2, 4, 6, and 8 weeks, respectively. The best results were obtained when synthetic seeds were stored at 4°C and germinated on MS medium. Regenerated plantlets were successfully established in potting soil.

## 1. Introduction

Recently, the use of alginate encapsulation of *in vitro* cultured shoot tips as an alternative to somatic embryos to develop synthetic seeds has increased [1, 2]. This increase in the use of microshoots in synthetic seed development is due to the fact that encapsulation of vegetative propagules offers an efficient and cost-effective system for clonal propagation of plant species [3, 4]. Moreover, most plant species are more readily amenable to *in vitro* shoot culture than to regeneration via somatic embryogenesis, which is, for the most part, highly species or genotype dependent. Synthetic seed technology can be used for several purposes. For example, it can be used in conjunction with micropropagation for *in vitro* conservation, germplasm storage, and reduction of the need for transferring and subculturing during off-season periods [5]. Cold storage of encapsulated or synthetic seeds has the potential to reduce the cost of maintaining germplasm cultures as well as to reduce the possibility of genetic instability that could result from frequent subculturing [5, 6]. The technology is particularly useful for the propagation of rare hybrids, elite genotypes, and genetically engineered plants whose seeds are either too expensive or not readily available [7].

Begonias are among the most popular ornamental plants in the world thanks to their large, showy, and long-lasting multicolor flowers, ranging from white to pink, red, and yellow. They are used as garden plants and potted plants, in hanging baskets, and as greenhouse flowers [8]. Begonias have also been used as potherbs or leaf vegetables in many parts of the world, and the roots and tubers of some species have been reported to possess antimicrobial activities and are used to treat various ailments [9–11]. Begonias are divided into three categories based on rootstocks: tuberous, fibrous, and rhizomatous. Tuberous and fibrous begonias are grown mostly for their flowers, whereas rhizomatous begonias are cultivated for their large, attractive foliage. As one the largest angiosperm genera containing about 2000 species [8] and with new species continuing to be discovered in various parts of the world [12–16], *Begonia* is a major component of the floriculture industry with a wholesale value of over $4 billion in 2011 statistics reported for 15 US states [17]. The main purpose of the current study was to establish an encapsulation method for *Begonia* microshoots as explants for short-term storage and germplasm exchange. Two *Begonia* semperflorens cultivars, Sweetheart Mix and BabyWing White, were used.

## 2. Materials and Methods

### 2.1. Plant Materials

Explants were excised from *in vitro* cultures of *Begonia* “Sweetheart Mix” and “BabyWing White” maintained on Murashige and Skoog (MS [18]) medium with 2% sucrose, 0.75 g/L MgCl_2_, and pH 5.8. All media were sterilized by autoclaving at 121°C and 1.1 kg/cm^2^ for 15 minutes after addition of 2 g/L Gelrite (Sigma-Aldrich, St. Louis, MO). Explants consisted of microshoots 4–8 mm long.

### 2.2. Encapsulation Procedure and Duration of Exposure to Calcium Chloride

**Figure 1:**
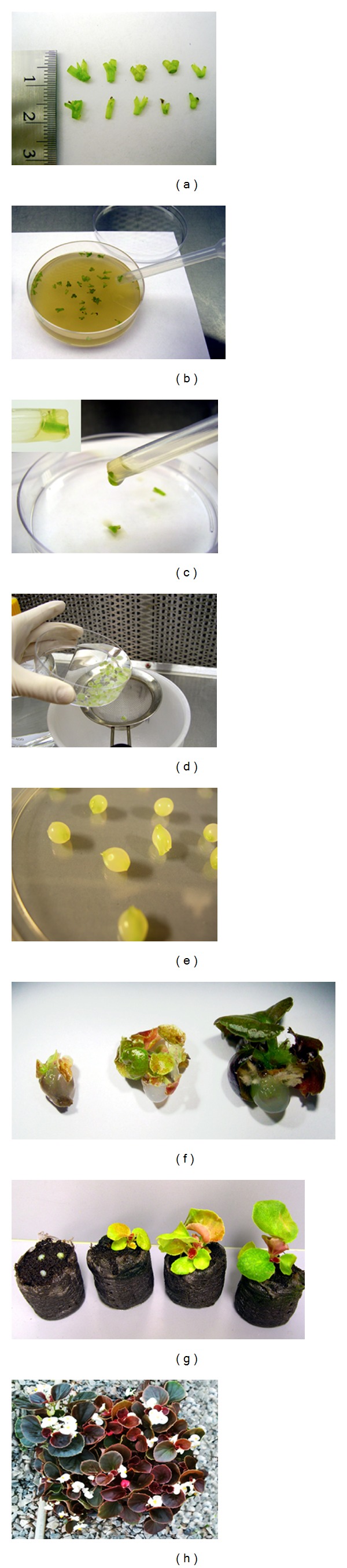
Encapsulation, germination, and maturation of *Begonia* microshoots. (a) Microshoot explants (4–8 mm long) were excised from *in vitro* cultures of *Begonia* cv. Sweetheart Mix and cv. BabyWing White. (b) Explants were submerged in sodium alginate solution for ~2 minutes and then individually suctioned into a sterile, disposable pipette with enough sodium alginate solution to encapsulate. (c) Each explant/sodium alginate combination was then released into a container of CaCl_2_·H_2_O and left for 30 or 45 minutes. (d) After the allotted time, the calcium chloride was drained from the newly formed synthetic seeds (encapsulated explants) by pouring the mixture into a sterile strainer. The synthetic seeds were rinsed at least three times with sterilized water to remove any remaining CaCl_2_·H_2_O. (e) Encapsulated explants or synthetic seeds were then placed in one of three container types: empty, sterile Petri dish (for storage), MS media, or peat-based substrate (PBS; Jiffy 7 peat pellets). (f) Typical growth pattern of synthetic seeds in MS media. (g) Typical growth pattern of synthetic seeds in soil. (h) Both MS media and PBS grown synthetic seeds developed into mature, thriving plants.

The steps involved in the scheme used for encapsulation, germination, and maturation of *Begonia* microshoots are shown in Figure 1. Synthetic seeds were formed using 3% sodium alginate in MS salt solution as the gel matrix and 100 mM calcium chloride (CaCl_2_·H_2_O) for complexation. Both the sodium alginate and calcium chloride solutions were sterilized by autoclaving at 121°C and 1.1 kg/cm^2^ for 15 minutes. Explants were submerged in sodium alginate solution for ~2 minutes. Explants, along with enough sodium alginate solution to encapsulate each one, were individually suctioned into a sterile, disposable pipette that had been modified by cutting the tip so that its diameter was ~7 mm.

During synthetic seed formation, the sodium alginate/explant combination was released into CaCl_2_·H_2_O and maintained for either 30 or 45 minutes to examine how duration in CaCl_2_·H_2_O might affect germination percentage. After the allotted time, the calcium chloride was drained from the newly formed synthetic seeds (encapsulated explants) by pouring the mixture into a sterile strainer. The synthetic seeds were rinsed at least three times with sterilized distilled water to remove any remaining CaCl_2_·H_2_O.

### 2.3. Storage Temperature and Duration

Two storage environments, 4°C refrigeration and room temperature (~22°C), and five storage durations (0, 2, 4, 6, and 8 weeks) were evaluated. Synthetic seeds as well as nonencapsulated explants (control) were placed in empty, sterile Petri dishes and stored under 4°C refrigeration or at room temperature (~22°C) for selected durations (0, 2, 4, 6, or 8 weeks). After the allotted storage period, the synthetic seeds and the control explants were placed in either MS media or peat-based substrate as described below. Germination percentages were monitored for all groups beginning with week 0 and extending through week 8.

### 2.4. *In Vitro* and *Ex Vitro* Germination

Two types of growth media, an *in vitro* germination medium and an *ex vitro* nonsterile peat-based substrate (PBS; Jiffy-7 Pellets, Shippegan, NB, Canada), were used. The culture medium consisted of Murashige and Skoog (MS) [18] medium supplemented with 2% sucrose, 0.75 g/L MgCl_2_ and adjusted to pH 5.8. All media were sterilized by autoclaving at 121°C for 15 minutes after addition of 2 g/L Gelrite. Synthetic seeds were planted in both MS medium and nonsterile PBS as parallel studies. The synthetic seeds that were plated on MS media were maintained at 22°C, 50% humidity, and 16-hour days under fluorescent lights with a photon flux averaging 154 *μ*moL m^−2^ s^−1^. Those in PBS were placed under grow lights and maintained at 23–25°C, 47–50% relative humidity, and 16-hour days under fluorescent lights with a photon flux of 71 *μ*mol m^−2^ s^−1^. A set of nonencapsulated explants served as a control and was maintained on the media described above and under the same environmental conditions. Germination, also referred to as conversion or regrowth by various authors [19–22], was defined as the point at which plant growth was observed outside of the synthetic seed matrix.

### 2.5. Experimental Design and Statistical Analysis

The experiment was conducted in a five-factorial randomized complete block design, with two media (MS medium and Jiffy 7 peat pellets), two cultivars (BabyWing White and Sweetheart Mix), three CaCl_2_·H_2_O exposure durations (0, 30, and 45 min), two storage temperatures (4 and 22°C), and five storage durations (0, 2, 4, 6, and 8 weeks). There were five replications for each treatment, with three microshoots per replication. The experiment was repeated twice. Data were collected as the count of shoots that “germinated” (developed successfully) in each experimental unit. Data were analyzed using generalized linear mixed models with the GLIMMIX procedure of SAS (version 9.3; SAS Institute Inc., Cary, NC) and the Poisson distribution. Models were evaluated beginning with a full model containing main factors and all interactions. Models were reduced by sequentially eliminating nonsignificant (*P* ≤ 0.05) interaction terms. The Schaffer-Simulated method was used for multiple mean comparisons.

Initial analyses determined that two factors, medium and storage duration, were highly influential in significant interaction terms; therefore, subsequent analyses were conducted individually by combination of medium and storage duration. Further analyses using cultivar, CaCl_2_·H_2_O exposure duration, and storage temperature determined that there were no significant interactions; therefore, final models contained only the three main effects. Means are presented as percentages.

## 3. Results and Discussion

Germination percentage of encapsulated as well as nonencapsulated (control) *Begonia* microshoots stored in two environments, namely, at 4°C and room temperature (~22°C) for various durations (0, 2, 4, 6, and 8 weeks) was evaluated. In both environments, *Begonia* shoot tips were dipped into 3% sodium alginate in MS salt solution as the gel matrix developed into nicely formed beads (hydrogels) and subsequently mature plants, irrespective of time of exposure (30 or 45 min) to the complexing agent, 100 mM CaCl_2_·H_2_O, or the growing medium, MS or PBS medium (Figure 1).

### 3.1. Effect of Cultivar on Germination

**Figure 2:**
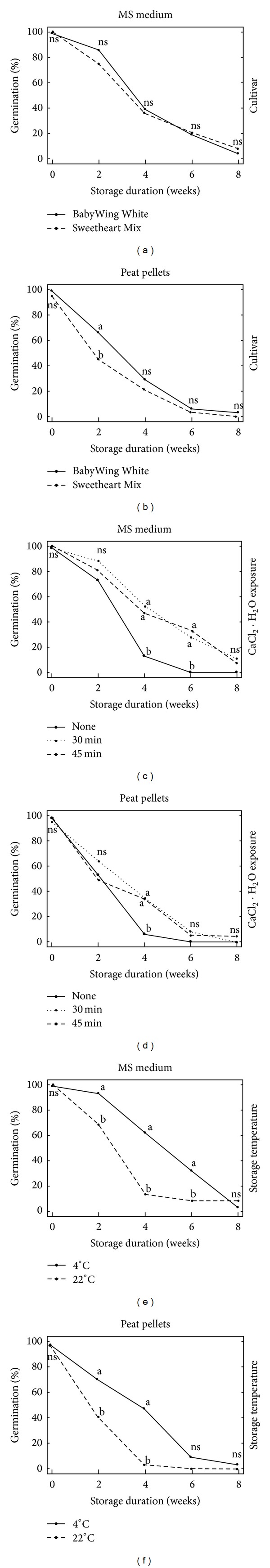
Percentages of encapsulated microshoots (synthetic seeds) and nonencapsulated microshoots of two *Begonia* cultivars “germinating” (developing successfully) after storage and subsequent transfer to substrate (MS medium (*in vitro*) or peat pellets (*ex vitro*)). Encapsulated microshoots were exposed to CaCl_2_·H_2_O after encapsulation, whereas nonencapsulated microshoots served as a control for this factor. Medium and storage duration were highly influential in significant interaction terms; therefore, analyses of cultivar ((a) and (b)), CaCl_2_·H_2_O exposure duration ((c) and (d)), and storage temperature ((e) and (f)) were conducted for each combination of medium and storage duration. Cultivar, CaCl_2_·H_2_O exposure duration, and storage temperature factors showed no significant interactions; therefore, means shown (dots) in each subfigure are averages over the other two factors. Means for a specific storage duration (vertical pairs or trios) within each figure are significantly different (*P* = 0.05) when labeled with “a” and “b,” or not significantly different when labeled with “ns.” The Schaffer-Simulated method was used for multiple mean comparisons.

There was no significant difference (*P* = 0.05) in germination percentages between synthetic seeds of the two cultivars, BabyWing White and Sweetheart Mix, grown in MS medium *in vitro* (Figure 2(a)). The results obtained under *ex vitro* conditions mirrored those achieved with MS medium, except that germination percentage for BabyWing White synthetic seeds in peat-based substrate was significantly higher (*P* = 0.05) than that obtained for Sweetheart Mix at week 2, with 66 and 45% germination percentage for BabyWing White and Sweetheart Mix, respectively (Figure 2(b)). In fact, germination percentage for BabyWing White was consistently higher, albeit not significantly, than that for Sweetheart Mix even after 8 weeks. For BabyWing White, germination percentages of 39% and 29% were obtained after 4 weeks when encapsulated shoots were grown in MS medium and peat-based substrate, respectively (Figures 2(a) and 2(b)). Similarly, for Sweetheart Mix, germination percentages of 36% and 21% were obtained after 4 weeks in MS medium and peat-based substrate, respectively. However, germination capacity for both cultivars decreased substantially with extended storage duration. Similar results have been reported by other workers [7]. In short, although low germination (3.3% for BabyWing White and 13.3% for Sweetheart Mix) could be achieved for synthetic seeds of both genotypes in an *in vitro* environment even at 8 weeks of storage duration (Figures 2(a) and 2(b)), similarly, encapsulation of explants improved germination under *ex vitro* conditions when Jiffy-7 pellets were used as nonsterile substrate (Figures 2(a) and 2(b)).

### 3.2. Effect of Exposure Duration of CaCl_**2**_·H_**2**_O and Encapsulation Matrix on Germination

The germination percentage was not significantly (*P* = 0.05) affected by the duration of exposure to calcium chloride whether the encapsulated seeds were maintained for 30 or 45 min in 100 mM CaCl_2_·H_2_O and whether MS medium or PBS was used (Figures 2(c) and 2(d)). Daud et al. [23] found that 30 min was the optimal exposure time for best germination percentage and that both shorter (10 min) and longer (90 min) exposure durations resulted in reduced germination of African violet (*Saintpaulia ionantha* Wendl.). On the other hand, Castillo et al. [24] reported that a relatively short (10 min) duration of exposure to CaCl_2_·H_2_O and only 2.5% alginate provided uniform encapsulation of embryos and the highest germination percentage (77.5%) of *Carica papaya* L. Successful production of synthetic seeds depends on several factors, including the concentration and type of gel needed for encapsulation of microshoots or somatic embryos, the duration of exposure of encapsulated seeds to CaCl_2_·H_2_O, and plant species [23, 25–28]. Several gel types are used for encapsulation, but the most commonly used matrix is sodium alginate because of low cost, gelling properties, and nontoxic nature [29]. The integrity or hardness of the hydrogels depends for the most part on the number of Na^+^ ions (in sodium alginate solution) exchanged with Ca^++^ ions (in CaCl_2_·H_2_O solution) resulting in the formation of insoluble calcium alginate [23]. In the current study, nicely formed beads were easily obtained with 100 mM CaCl_2_·H_2_O and 3% sodium alginate. Our results were in agreement with those of Daud et al. [23] who reported that an encapsulation matrix of 3% alginate with 100 mM CaCl_2_·H_2_O was ideal for the formation of *Saintpaulia ionantha* Wendl. microshoot beads as higher concentration of sodium alginate (4-5%) resulted in beads that were too hard, causing a germination delay, while lower concentration of sodium alginate (1-2%) produced beads that readily burst because they were too fragile and difficult to handle. Indeed, a 3% sodium alginate appears to be the optimum concentration for a great number of species as low concentrations (1-2%) result in beads too soft to handle and higher concentrations (≥4%) in beads too hard, preventing the emergence of shoots and roots [29–33]. On the other hand, germination percentage was significantly affected by encapsulation as marked improvement was achieved in germination percentage for encapsulated microshoots compared with nonencapsulated explants in both *in vitro* and *ex vitro* environments (Figures 2(c) and 2(d)). The germination percentage of encapsulated microshoots, whether exposed to CaCl_2_·H_2_O for 30 or 45 min, was consistently higher than that of nonencapsulated microshoots up to 6 and 4 weeks in MS medium and PBS, respectively. These improved germination percentages using synthetic *Begonia* seeds are in agreement with other findings from other workers for several other species including *Camellia *spp., *Zingiber officinale*, and *Ruta graveolens* [20, 21, 34].

### 3.3. Effect of Storage Temperature and Substrate on Germination

Storage temperature significantly (*P* = 0.05) affected germination frequency in both *in vitro* and *ex vitro* environments as germination percentage was significantly higher for encapsulated microshoots stored at 4°C (up to 6 weeks) than for those grown at 22°C (up to 4 weeks). Germination percentage of encapsulated microshoots in both growing environments declined over time, but this decrease was more pronounced when explants were stored at room temperature than under refrigeration (Figures 2(e) and 2(f)). For example, in MS medium, germination percentage was still relatively high at 32% after 6 weeks, whereas only 8% germination was obtained at room temperature at the same time point (Figure 2(e)). Similarly, in PBS, germination percentages after 4 weeks were 47% and 3% for 4 and 22°C, respectively (Figure 2(f)). In other words, germination was almost 16-fold higher when encapsulated explants were stored at low temperature. It is noteworthy that, even at room temperature and under nonsterile conditions, 41% of synthetic seeds still germinated after 2 weeks (Figure 2(f)).

**Figure 3:**
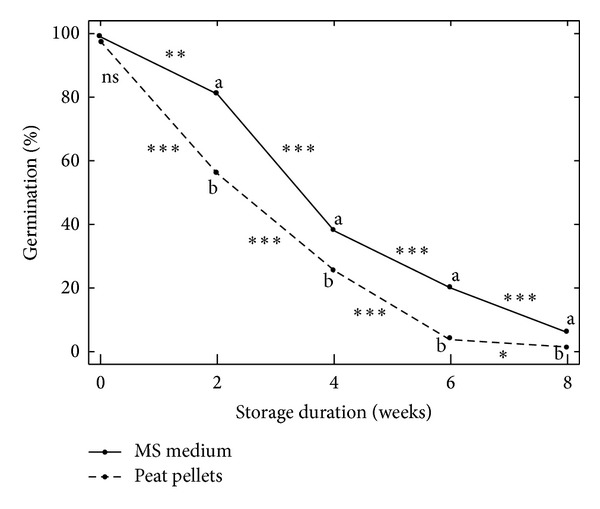
Percentages of encapsulated microshoots (synthetic seeds) and nonencapsulated microshoots of two *Begonia* cultivars “germinating” (developing successfully) after storage and subsequent transfer to MS medium (*in vitro*) or peat pellets (*ex vitro*). Medium and storage duration were highly influential in significant interaction terms among five treatment factors; therefore, analyses were run at each combination of these two factor levels. Means shown (dots) are averages among the three additional treatment factors (cultivar, CaCl_2_·H_2_O exposure duration, and storage temperature), which showed no significant interaction effects when analyzed by medium and storage duration. Means for the two media at a specific storage duration (vertical pairs) are significantly different (*P* = 0.05) when labeled with “a” and “b” or not significantly different when labeled with “ns.” Means for storage durations connected by a line segment are significantly different at *P* = 0.05 (*), 0.01 (**), or 0.001 (***). The Schaffer-Simulated method was used for multiple mean comparisons.

Overall, significantly better germination percentages were obtained over time when the encapsulated seeds were grown in MS medium *in vitro* than in peat pellets *ex vitro* (Figure 3). Furthermore, the improved germination percentage of encapsulated microshoots over control explants, even after a considerable amount of storage time, can be attributed to the inclusion of MS salts in encapsulation matrix, which serves as an artificial endosperm to the encapsulated microshoots for conversion to plantlets [35, 36]. Hung and Trueman [37] reported that direct transfer of synthetic seeds of African mahogany (*Khaya senegalensis*) to nonsterile substrates was ineffective unless the seeds were allowed to preconvert (i.e., to form roots *in vitro* with the aid of growth regulators) prior to transfer as most of the seeds were contaminated with fungi within a week. However, in the current study, such an extra step was not required as synthetic *Begonia* seeds readily germinated, formed roots, and were easily acclimated when transferred to nonsterile peat-based media (Figure 1(g)).

## 4. Conclusions

Production of synthetic seeds of both *Begonia* cultivars, BabyWing White and Sweetheart Mix, using the protocol developed in this study was relatively easy. Encapsulated seeds kept at low temperature (4°C) could be stored for a longer period of time and had a higher germination percentage than those stored at room temperature (~22°C). Also, better germination percentages were obtained over time when the encapsulated seeds were grown in MS medium than in PBS, irrespective of the storage environment (4°C or 22°C). However, storage at room temperature has the advantage of avoiding costs associated with refrigeration equipment. Seeds of Sweetheart Mix are actually a mixture of seeds from different genotypes, so the fact that synthetic seeds of this cultivar could be easily formed and germinated using our protocol suggests that this procedure could be applied for an efficient production of synthetic seeds from microshoots of other *Begonia* species and cultivars.

## Abbreviations

cv.: Cultivar
MS: Murashige and Skoog medium
PBS: Peat-based substrate.
